# Multiple Grasp-Specific Representations of Tool Dynamics Mediate Skillful Manipulation

**DOI:** 10.1016/j.cub.2010.01.054

**Published:** 2010-04-13

**Authors:** James N. Ingram, Ian S. Howard, J. Randall Flanagan, Daniel M. Wolpert

**Affiliations:** 1 Department of Engineering, University of Cambridge, Trumpington Street, Cambridge CB2 1PZ, UK; 2 Department of Psychology and Centre for Neuroscience Studies, Queen's University, Kingston, ON K7L 3N6, Canada

**Keywords:** SYSNEURO

## Abstract

Skillful tool use requires knowledge of the dynamic properties of tools in order to specify the mapping between applied force and tool motion [1–3]. Importantly, this mapping depends on the orientation of the tool in the hand. Here we investigate the representation of dynamics during skillful manipulation of a tool that can be grasped at different orientations. We ask whether the motor system uses a single general representation of dynamics for all grasp contexts or whether it uses multiple grasp-specific representations. Using a novel robotic interface [4], subjects rotated a virtual tool whose orientation relative to the hand could be varied. Subjects could immediately anticipate the force direction for each orientation of the tool based on its visual geometry, and, with experience, they learned to parameterize the force magnitude. Surprisingly, this parameterization of force magnitude showed limited generalization when the orientation of the tool changed. Had subjects parameterized a single general representation, full generalization would be expected. Thus, our results suggest that object dynamics are captured by multiple representations, each of which encodes the mapping associated with a specific grasp context. We suggest that the concept of grasp-specific representations may provide a unifying framework for interpreting previous results related to dynamics learning.

## Highlights

► Tools can be used in different contexts, such as the grasped orientation ► We ask whether tools have a single, general, or multiple context-specific representations ► Subjects manipulated a virtual tool simulated by a novel robotic interface ► Results show that tools engage multiple grasp-specific representations of dynamics

## Results

**Figure 1:**
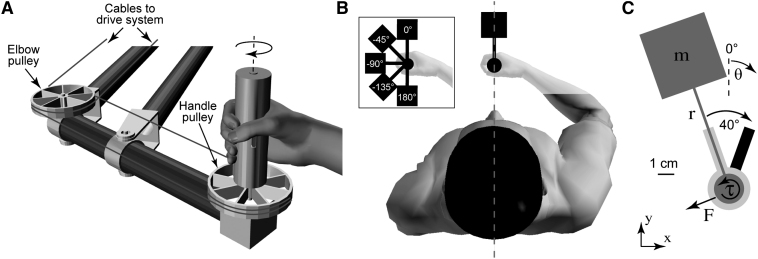
Robotic Manipulandum and Virtual Tool Manipulation Task (A) The WristBOT is a planar two-dimensional robotic manipulandum that includes torque control at the vertical handle. Cables and pulleys (two are shown) implement the transmission system between the handle and drive system at the rear of the manipulandum (not shown). A safety cover that encloses the handle pulley and cables has been removed for clarity. (B) Top view of subject showing visual feedback of a virtual tool, which is projected over the subject's hand in the plane of movement. Visual feedback (see C) is consistent with grasping the tool at its base. In reality, subjects grasp the vertical handle of the WristBOT, which is aligned with visual feedback. The WristBOT handle translates in the horizontal plane (x and y) and rotates around the vertical axis. Subjects view visual feedback in a mirror that prevents them from seeing either their hand or the manipulandum. Dotted line shows subject's midsagittal plane, which is aligned with the hand and the vertical rotation axis of the tool. Inset shows top view of subject's hand overlaid with five different visual orientations of the tool. (C) Virtual tool dynamics were simulated as a point mass (mass, m) on the end of a rigid rod (length, r) of zero mass (see Supplemental Experimental Procedures). Visual feedback of the tool (dark gray figure) was provided and updated in real time. Subjects grasped the tool by the circular handle, which was aligned with their hand. The task involved rotating the tool 40° from a starting angle (light gray bar) to a target angle (black bar) while maintaining the handle within a circular home region (light gray). Rotation generated translational forces (F) and rotational torques (τ) at the handle. Figure shows a grayscale version of actual visual feedback presented to subjects (scale bar represents 1 cm). Annotations have been added.

Subjects rotated a virtual hammer in the horizontal plane by grasping and rotating the vertical handle of a novel robotic manipulandum (the WristBOT [4]; Figure 1A). The WristBOT can produce forces and torques that depend on the position and orientation of the handle. Visual feedback of the hammer was projected over the subject's hand (Figure 1B) and updated in real time. The dynamics of the hammer were simulated as a point mass on the end of a rigid rod (Figure 1C; see also Supplemental Experimental Procedures available online for full details).

Trials were performed in pairs in which the hammer was first rotated 40° counterclockwise (CCW) and then 40° clockwise (CW) between two visually presented targets. The targets were oriented bars emanating from the central disc representing the home position (Figure 1C). The orientation of the hammer and the targets could be varied in order to present the tool at different orientations (inset of Figure 1B; see also Figure S1). Subjects were asked to keep the handle stationary within the home position as they rotated the tool. Such pure rotation required subjects to generate time-varying torques and forces in the horizontal plane. For a given angular velocity profile, the direction of the force vector depends only on the orientation of the hammer, whereas the force magnitude depends on the mass and rod length.

The aim of the first experiment was to determine whether subjects could recall the general structure of the dynamics based purely on vision of the hammer. Specifically, we examined whether subjects generated forces in the appropriate direction when manipulating hammers grasped at different orientations relative to the hand. The position of the handle was held fixed by a simulated spring, and therefore subjects did not experience kinematic errors (translation of the handle) that might trigger reactive forces and learning. These error-clamp trials allowed us to measure the anticipatory forces produced by subjects.

**Figure 2:**
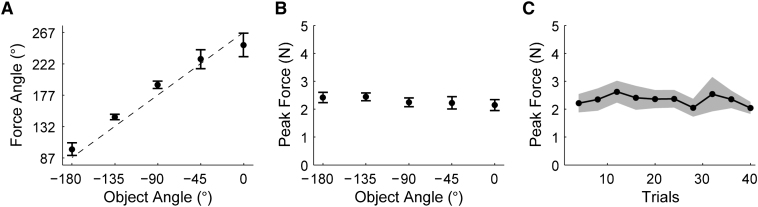
Tool Dynamics Cued by Visual Feedback (A) Direction of peak anticipatory forces as a function of visual orientation of the tool. Data points are circular means (±1 circular standard error [SE]) across subjects (n = 8). Dotted line shows force direction that would fully compensate for the tool dynamics at that orientation (based on simulations; see Results). (B) Peak magnitude of anticipatory forces as a function of visual orientation of the tool. Data points are means (±1 SE) across subjects (n = 8). (C) Peak magnitude of anticipatory forces across experimental blocks. Each data point is the mean of 4 trials averaged across subjects. Shaded region is ±1 SE.

The hammer was presented at five orientations (inset of Figure 1B), and subjects performed four trial pairs at each orientation. Despite having no training with the perturbing dynamics associated with rotating the tool, subjects generated substantial translational forces during the rotation. To assess the relation between the visual orientation of the hammer and the direction of the forces (Figure 2A), we calculated the strength (ρ) of the circular-circular linear association [5] for each subject. There was a significant relation (mean ρ = 0.47 [CW] and 0.26 [CCW] across subjects, both p < 0.001) between the hammer's visual orientation and the force direction, with offsets of −86.8° ± 30.8° (circular mean ± circular standard deviation [SD]) and 81.2° ± 17.4° for CW and CCW rotations, respectively. Simulations demonstrate that for a pure rotation of a hammer about the handle (assuming a Gaussian angular velocity profile), this offset should be −93° (or 93°) for CW (or CCW) rotations. These results show that, given only vision of the hammer, subjects can recall the appropriate dynamic structure, allowing them to predict the force direction required for each orientation.

In contrast to the orientation-dependent modulation of force direction, subjects did not modulate peak force magnitude either for the different visual orientations of the hammer (analysis of variance [ANOVA], p = 0.53; Figure 2B) or across successive blocks of four trials (ANOVA, p = 0.76; Figure 2C). Moreover, peak force magnitude was similar across subjects (2.3 ± 0.1 N; mean ± standard error) and was, on average, appropriate for a hammer head mass of 444 g. It is possible that subjects estimated the required force magnitude based on the visual size of the mass and the rod length, both of which were constant across trials. In general, however, direct sensorimotor experience is required to learn the dynamic parameters that specify force magnitude [6–10].

In the second experiment, we examined how experience with the dynamics of a specific hammer, confined to a single orientation, generalized to other orientations. The aim was to test two alternative hypotheses. We asked whether the motor system uses multiple representations of the dynamics associated with different tool orientations or a single general representation applied to all orientations. If a single general representation exists, single context learning should lead to perfect generalization in novel contexts. In contrast, limited generalization to novel contexts would suggest the existence of multiple context-specific representations.

Subjects performed multiple blocks of 90 trials. They first rotated the hammer at a training orientation (0°) for 60 trials while experiencing full dynamics (torques and forces). Subjects then performed an additional 30 trials consisting of 24 training trials (full dynamics at 0°) and 6 randomly selected error-clamp trials: 3 at the training orientation (0°) and 3 at a transfer orientation (−90°). Although the visual orientation of the hammer (the grasp context) could change between trials, the orientation of the hand and arm and the required rotation were kept constant. Within a given block, the hammer head mass was 0.7%, 1.0%, or 1.3% of the subject's body mass.

**Figure 3:**
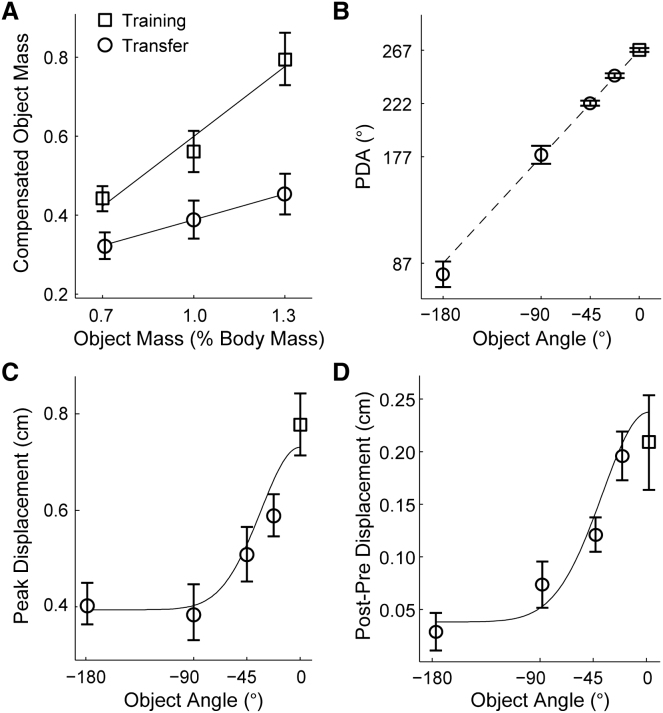
Performance after Training at a Single Orientation and Subsequent Transfer to Novel Orientations (A) Anticipatory forces during probe trials (expressed as compensated object mass) at the training orientation (0°, squares) and transfer orientation (−90°, circles) for tools of different mass. Tool mass is expressed as percentage of each subject's body mass. Compensated object mass is the tool mass (also expressed as percentage body mass) for which the forces would have been appropriate. Data points are means (±1 SE) across subjects (n = 9). Lines show average of individual linear regressions across subjects. (B) Peak handle displacement angle (PDA) during probe trials as a function of visually presented orientation of the tool. Dotted line shows the direction that would fully compensate for the tool dynamics (plotted as in Figure 2A). Data points are circular means (±1 circular SE) across subjects (n = 8) at the training orientation (0°, square) and transfer orientations (−22.5°, −45°, −90°, 180°, circles). (C) Peak handle displacement (independent of direction) during probe trials that shows the transfer of training as a function of the visually presented orientation of the tool. Data points are means (±1 SE) across subjects (n = 8) at the training orientation (0°, square) and transfer orientations (−22.5°, −45°, −90°, 180°, circles). The orientation-dependent decrease in displacement was fit by a half Gaussian for each subject, and the line shows the average fit across subjects (mean fit standard deviation [SD] = 34°). (D) Increases in peak displacement following probe trials that show effects of partial deadaptation as a function of visually presented orientation of the tool, plotted as in (C). Values are means of the subject-by-subject difference between preprobe and postprobe displacements at the training orientation (0°) after partial deadaptation with probe trials at different visually presented orientations of the tool. As in (C), the orientation-dependent decrease in displacement was fit by a half Gaussian for each subject, and the line shows the average fit across subjects (mean fit SD = 39°).

We measured peak force magnitude on error-clamp trials and estimated the hammer mass for which this force would have been appropriate had the handle not been error clamped. We termed this value the compensated object mass. At the training orientation (0°), the compensated object mass scaled with the mass of the hammer (Figure 3A, squares), showing that subjects adapted their force output based on sensorimotor experience. However, limited generalization of this adaptation was observed when the hammer was presented at −90° (Figure 3A, circles). Specifically, the compensated object mass at the transfer orientation was approximately half of that observed at the training orientation. There was a significant positive relation between the experienced and the compensated object mass across subjects for both the training (slope = 0.59 ± 0.17; t test, p < 0.001) and transfer (slope = 0.22 ± 0.15; t test, p < 0.005) orientations. The slope for training was significantly steeper than for transfer (t test, p < 0.005). These results indicate that subjects were not simply using a default force but were representing the inertial properties of the tool that they experienced at the training orientation. The limited transfer suggests that the representation was orientation specific, consistent with the multiple representation hypothesis.

It should be noted that even at the training orientation, the compensated object mass was around 60% of the true mass. As such, the force generated by subjects would not have fully compensated for the tool dynamics. However, this is expected because error-clamp trials probe only the anticipatory (feedforward) forces and do not elicit any reactive (feedback) forces that would normally complement predictive compensation [11].

In the third experiment, we examined transfer to a range of orientations. Subjects first rotated the hammer at 0° for 64 trials while experiencing full dynamics. They then performed 15 blocks of 26 trials as follows: the first 8 trials of each block were presented at one of five transfer orientations (0°, −22.5°, −45°, −90°, or −180°) randomly selected, with the forces turned off. Peak handle displacement was measured during these probe trials as an indicator of the forces produced by subjects. This allowed us to examine the generalization of adaptation as a function of the visual orientation of the tool. In addition, presenting a small number of zero-force probe trials causes partial deadaptation of the learned force magnitude, allowing us to examine the generalization of deadaptation. The last 18 trials of each block were presented at the training orientation (0°). The first 2 trials immediately following the probe trials were error-clamp trials, during which anticipatory forces were measured. The final 16 trials were once again under full dynamics of the tool. Subjects completed 15 blocks, which included three presentations of each transfer orientation and each direction of rotation (CW or CCW).

Consistent with results from the first experiment, the angle of the peak displacement during probe trials varied with hammer orientation (Figure 3B) and was close to the direction predicted by the dynamics. The peak displacement magnitude was also measured in order to quantify generalization. If subjects generalize perfectly to a particular transfer orientation and thus generate the appropriate force at the handle, the displacement should be as large as at the training orientation. However, the peak displacement on probe trials decreased progressively as the transfer orientation increased relative to the training orientation (Figure 3C), consistent with the multiple representation hypothesis. The pattern of orientation-dependent generalization was well fit by a half Gaussian (mean fit SD = 34°).

In addition to the orientation-dependent decrease in displacement observed during probe trials, we found similar orientation dependence for the increase in peak displacement immediately following probe trials, when the tool returned to the training orientation (Figure 3D). This increase in displacement can be understood as resulting from partial deadaptation following the zero-force probe trials. Probe trials near or at the training orientation caused the greatest deadaptation, as expected from the multiple representation hypothesis. This pattern of orientation-dependent deadaptation could also be fit by a half Gaussian (mean fit SD = 39°).

It is important to note that the generalization seen here is distinct from that reported previously. Previous studies have examined generalization by exposing subjects to novel dynamics (e.g., a state-dependent force field) in one kinematic context (e.g., a region of the workspace or direction of movement) and testing generalization in a second kinematic context, where the dynamics have not been experienced (e.g., [12, 13]). In contrast, in the current study, the kinematic context is kept constant. That is, the position and orientation of the arm and hand are fixed, as is the movement required at the hand. The only factor that changes is the visual orientation of the tool. As such, we are specifically probing the representation of the tool rather than the representation of the arm.

**Figure 4:**
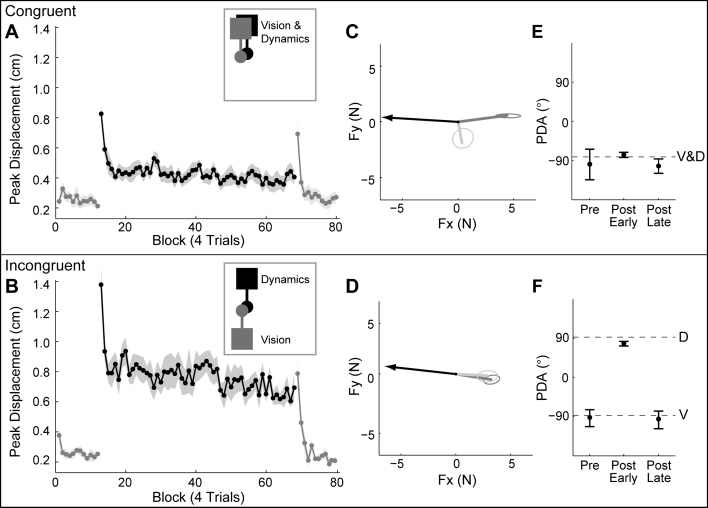
Performance during Exposure when Vision is Either Congruent or Incongruent with Dynamics (A) Peak handle displacement (independent of direction) averaged across subjects (n = 7) for each block (4 trials) for a visually congruent tool at 0° (see inset; a second group experienced a visually congruent tool at 180°). Shaded region is ±1 SE. Black trace shows performance during exposure to full dynamics. Gray traces show pre- and postexposure phases with no translational forces. (B) Peak displacement, plotted as in (A), for an incongruent tool with a visual orientation of 180° (0° for dynamics; see inset; a second group experienced an incongruent tool with vision at 0° and dynamics at 180°). (C) Peak anticipatory force vectors for a congruent tool at 180°. Black arrow shows circular mean (across subjects) for peak forces produced by the dynamics of the tool. Dark and light gray lines show circular mean (across subjects) for peak anticipatory forces at the training (dark gray) and −90° transfer (light gray) orientations. Ellipses show 99% confidence intervals across subjects. (D) Peak anticipatory force vectors for an incongruent tool at 180°, plotted as in (C). (E) PDA during entire pre-exposure phase (48 trials) and for early (first 12 of 48 trials) and late (last 12 of 48 trials) stages of postexposure (deadaptation) phase, for the congruent tools. Dotted line shows PDA predicted from congruent vision and dynamics (V&D) of tool. Data points are circular means (±1 circular SE) across subjects. (F) PDA for the incongruent tools, plotted as in (E). Dotted lines show separate PDA predicted from dynamics (D) and vision (V) of the incongruent tools.

Previous studies of force adaptation when lifting objects have shown that subjects rely on visual cues but can, through sensorimotor experience, override these cues when they are misleading [8, 14, 15]. To further examine the interaction between vision and sensorimotor experience, in the fourth experiment we dissociated the visual orientation of the hammer from the orientation of its dynamics. The visual orientation was either congruent with the dynamics or incongruent. In the latter incongruent case, the visual feedback was rotated 180° relative to the dynamics. For both congruent and incongruent conditions, one group of subjects experienced the visual hammer at 0° and another at 180° (making four groups). In the first phase of the experiment, subjects rotated the hammer with the forces turned off for 24 trials. As in the first experiment, subjects generated a small force in the appropriate direction based on the visual cue, resulting in a small initial displacement of the handle (Figures 4A and 4B). Subjects then performed a further 248 trials with full dynamics (Figures 4A and 4B). The initial displacement was larger in the incongruent condition, because initially subjects produced a force appropriate for the visual orientation and therefore opposite to that needed to compensate for the dynamics. Although the incongruent group showed significant learning, their final displacement (average of the last 18 trials) was significantly larger (t test, p < 0.001) than that of the congruent group. This larger displacement was associated with significantly smaller anticipatory forces (congruent: 3.7 ± 0.9 N; incongruent: 2.6 ± 0.6 N; t test, p < 0.005).

To evaluate the generalization of congruent and incongruent dynamics, subjects then performed 256 trials with full dynamics during which 1 error-clamp trial was inserted randomly every 8 trials, either at the training orientation (0° or 180°) or at a transfer orientation (−90°). Figures 4C and 4D show the force vectors produced on error-clamp trials at the training (dark gray) and transfer (light gray) orientations. For both groups, the forces produced by subjects on error-clamp trials at the training orientation (Figures 4C and 4D, dark gray vectors) were in the appropriate direction to compensate for the dynamics. For the congruent group, when the visual orientation was rotated to −90°, the force generated also rotated appropriately (Figure 4C, light gray vector). This is consistent with results of the first experiment showing that subjects produce forces that are appropriate for the visual orientation of the tool. In contrast, for the incongruent group, when the visual orientation rotated to −90°, the force generated by the subjects did not change. Specifically, for the congruent groups, the change in force direction did not differ significantly from 90° (t test, p = 0.64), whereas for the incongruent groups, it did not different significantly from 0° (t test, p = 0.60). Consistent with the second experiment, in the congruent group, force magnitude decreased significantly (t test, p < 0.001) by 57% ± 7% from the training to the transfer orientation (compare lengths of dark and light gray force vectors in Figure 4C). In contrast, no difference in force magnitude (t test, p = 0.49) between the training and transfer orientations was observed in the incongruent group (compare lengths of dark and light gray force vectors in Figure 4D).

These results show that when vision was incongruent with dynamics, the pattern of generalization was markedly different. Results for the congruent group, as with the previous three experiments, were consistent with the multiple representation hypothesis. However, for incongruent dynamics, neither force direction nor magnitude was modulated by the visual orientation of the tool, suggesting that a single representation was applied in the incongruent case.

Finally, because performance remained worse in the incongruent group relative to the congruent group (even after extensive experience), it is possible that the subject's preexisting representation of congruent dynamics was affecting their ability to represent the incongruent dynamics. Analysis of the direction of anticipatory forces during the postexposure phase provides further support for this possibility (compare Figures 4E and 4F). In the postexposure phase, subjects deadapted because the forces associated with tool dynamics were turned off (see postexposure gray trace in Figures 4A and 4B). Immediately following exposure in the incongruent condition, the direction of anticipatory forces was consistent with the incongruent dynamics (Figure 4F, “Post Early”). At the end of the postexposure phase, however, the direction of anticipatory forces had spontaneously reverted to be consistent with vision (Figure 4F, compare “Pre Late” and “Post Late”). This occurred even though subjects in the incongruent group had not experienced congruent dynamics during any stage of the experiment, suggesting that the representation of incongruent dynamics was labile relative to the representation of congruent dynamics.

## Discussion

When first rotating a hammer-like tool, subjects generated anticipatory forces in directions that were appropriate for its visual orientation. This indicates that subjects had prior knowledge of the structural form of the dynamics of the tool that could be recalled based on visual information alone. Previous studies have shown that visual information can be used to identify the location of the center of mass of an object [16] and that this information can be used to appropriately scale forces when lifting objects [17–20]. Visual information can also facilitate the sudden change in dynamics associated with grasping and releasing an object [21]. The current study shows that visual information can be used to recall complex grasp-dependent dynamics, such as those associated with rotating a hammer at different orientations.

When subjects experienced the full dynamics of the hammer at a particular orientation, they quickly scaled the magnitude of their anticipatory forces to the mass of the hammer. Thus, subjects started with a representation of the structural form of the dynamics and subsequently parameterized this representation following interaction with a specific hammer. We considered two mechanisms by which these dynamics are represented. In the single general representation model, the motor system would use a single context-invariant representation of dynamics that is transformed based on the visual orientation of the tool. In the multiple representation model, the motor system would use multiple context-specific representations for different tool orientations. Our results support the latter multiple representation model. Specifically, we found that adaptation to the dynamics of the hammer at one orientation showed limited generalization to novel grasp contexts in which the orientation of the hammer was changed relative to the hand. This is consistent with multiple representations, because a single representation would predict perfect performance at all orientations following exposure at a single orientation.

It has been suggested that the brain could use multiple internal models for sensorimotor control, with appropriate models being selected based on the context of the movement [22–24]. Our results are consistent with this framework, in which the sensorimotor control of tool use is mediated by multiple context-specific internal models, with the contribution of different models being smoothly modulated by the context, such as the visual orientation of the tool.

Although the current study focuses on dynamics that are familiar, many previous studies have examined how novel dynamics are represented [12, 13, 25–28]. Several of these studies conclude that novel dynamics, applied to the hand via a grasped handle, are represented in joint-based coordinates. This conclusion is based on patterns of generalization when the arm is rotated. Whereas good generalization is observed when the force field is rotated with the arm, poor generalization is seen when the orientation of the force field is held constant in Cartesian space [12, 26, 29]. However, a joint-based representation seems surprising given that subjects attribute force field dynamics to the grasped object rather than the arm [3, 21, 30–32]. Our results suggest an object-centered representation of the dynamics associated with novel force fields. Maintaining the orientation of the force field in Cartesian space when the arm is rotated is equivalent to changing the orientation of the tool relative to the hand. However, in the absence of visual feedback of tool orientation, subjects may assume that a grasped tool rotates with the arm. This would maintain a constant orientation relative to the hand. As such, the poor generalization observed in previous studies would be expected. Even if visual feedback of the force field orientation could be provided, our results, suggesting that the motor system learns grasp-specific models, would predict poor generalization. Rotating the force field with the arm is equivalent to maintaining the orientation of a grasped tool constant relative to the hand. Thus, the good generalization that is observed in this situation is consistent with the idea that the central nervous system learns grasp-specific models of tool dynamics.

Our results can be related to hypotheses regarding the mechanisms of visual object recognition. Specifically, it has been suggested that the visual system uses either single or multiple representations to solve the problem of viewpoint invariance for object recognition [33–35]. Evidence for multiple viewpoint-specific representations comes from studies in which subjects learn to visually recognize novel objects. When first trained with a novel object at a single orientation, subjects learn to recognize the object with progressively smaller reaction times until a minimum is achieved [36]. However, when subsequently presented with the same object at a novel orientation, reaction times increase monotonically with further departures from the training orientation. With additional training at novel orientations, reaction times again decrease. These results suggest that when learning to recognize a novel object, the visual system accumulates progressively more viewpoint-specific representations [37]. Our results suggest that a progressive accumulation of multiple context-specific representations may also occur when the motor system learns tool dynamics.

In summary, we have used a novel robotic manipulandum to show that subjects have an existing representation of the complex dynamics associated with hammer-like tools. The representation can be appropriately recalled by simple visual information, which captures the geometric features of the tool. It is locally parameterized based on experience with a specific tool, with limited generalization to orientations where the tool has not been directly experienced. These results suggest that our ability to use tools relies on multiple context-specific representations of dynamics rather than a single context-invariant representation.

## Supplemental Information

Document S1. Supplemental Experimental Procedures and Two Figures
