# Supplementary material for: Multiple Grasp-Specific Representations of Tool Dynamics Mediate Skillful Manipulation
Source: Curr Biol. 2010 Apr 13;20(7):618–23. doi: 10.1016/j.cub.2010.01.054 (PMC3501566; doi:10.1016/j.cub.2010.01.054)
Supplement: Document S1. Supplemental Experimental Procedures and Two Figures [file mmc1.pdf]

Current Biology, Volume *20*

Supplemental Information

Multiple Grasp-Specific Representations of Tool Dynamics Mediate Skillful Manipulation

James N. Ingram, Ian S. Howard, J. Randall Flanagan, and Daniel M. Wolpert

Supplemental Experimental Procedures

Subjects (n=53) provided informed consent before participating and the study was approved by the local ethics committee. They were seated at a virtual reality system that could provide visual and haptic feedback of a simulated virtual object. They grasped the handle of a planar robotic manipulandum with their right hand. The novel, custom-built manipulandum (the WristBOT [1]; Figure 1A in the main text), could generate both translational forces in the horizontal plane and a rotational torque about the vertical handle. The task was to grasp the virtual object by its handle and rotate it back and forth between visual targets (Figure S1; see also Figure 1B in the main text). The object was a small hammer-like tool that consisted of a mass on the end of a rigid rod (Figure 1C in the main text). Subjects grasped the tool by the handle at the base of the rod with a comfortable posture, so that the wrist was near the midpoint of its range of motion. They maintained this posture throughout the experiment. They were told that the tool might wobble during the rotation and that they should try to maintain the handle as still as possible within the central home region.

The visual orientation of the tool could be varied between trials. For a given tool orientation, subjects performed trials that alternated between counter-clockwise (CCW) and clockwise (CW) rotations between two targets (Figure S1). The two targets where ±20º relative to the central orientation at which the tool was presented. Each trial thus required a movement of 40º amplitude. A trial began with the handle of the tool stationary within the home region and the rod aligned with either the +20° target (for CCW movements; Figure S1 A) or the -20° target (for CW movements; Figure S1, D). The onset of movement was then cued by a tone and the appearance of the second target (Figure S1, B and D). The trial ended when subjects had rotated the tool to reach the second target (Figure S1, C and F). Subjects were required to make the movement within 400 ms. They were warned if they took longer and had to repeat the trial if the movement exceeded 500 ms. Rest breaks (30-60 s) were given every 3-5 minutes.

Visual feedback of the tool was provided by a virtual reality projection system that overlaid computer generated graphics in the plane of the movement as previously described [1]. The visual tool (Figure S1; see also Figure 1C in the main text) consisted of a circular handle (radius 0.5 cm) attached to a 4 cm square mass by an 8 cm rod (width 0.2 cm). The home region was a

1 cm radius disc and targets were oriented rectangles (0.6 by 2.5 cm). The position and orientation of the WristBOT handle determined the position and orientation of the virtual tool.

Tool Dynamics

The WristBOT simulated the dynamics of the tool (see Figure 1C in the main text) as a point mass (m) at the end of a rigid rod (length r). When rotating the tool, subjects experienced a torque (τ) which depends on the angular acceleration ($\ddot{\theta}$) of the handle.

$$\tau = -mr^2\ddot{\theta} \quad (1)$$

Subjects also experienced a force at the handle which consisted of two orthogonal components. The first component was associated with the tangential force (F_T), which depends on the angular acceleration of the handle.

$$F_T = mr\ddot{\theta} \quad (2)$$

Note that F_T acts in the direction of the tangential acceleration of the mass, and is thus perpendicular to the rod.

The second component was associated with the centripetal force (F_C), which depends on the angular velocity ($\dot{\theta}$) of the handle.

$$F_C = mr\dot{\theta}^2 \quad (3)$$

Note that F_C acts perpendicular to the tangential velocity of the mass (towards the centre of rotation), and is thus parallel to the rod.

The resultant force vector (F) experienced by the subject at the handle is thus given by

$$\mathbf{F} = R(\theta)(-F_T \quad F_C)^T \quad (4)$$

where F is the two-dimensional force vector (in the coordinate system of the WristBOT, as specified in Figure 1C in the main text), θ is the angle of the rod (0° is aligned with the y-axis) and $R(\theta)$ is a 2 x 2 clockwise rotation matrix. Simulations demonstrated that pure rotation of the tool about the handle with a smooth (Gaussian) velocity profile gives rise to a force at the handle whose magnitude peaks soon after the peak in acceleration, with a direction of the peak force at -73° or +73° relative to the starting angle of the tool for CW and CCW rotations, respectively.

To avoid the need to compute angular velocity and acceleration, the dynamics were approximated by simulating the point mass attached to the end of the rod by a stiff spring (3000 N/m). Translation and rotation of the tool caused the spring to stretch which then generated forces and torques on the handle. At the same time, these forces were used to update the state of the simulated mass that included damping to prevent oscillations (7 N m-1 s). Simulations in Matlab (R14, The MathWorks Inc., Natick, MA, USA) verified that this approximation captured the dynamics of the tool very well.

For movements with the same rotational kinematics, both the torque and force magnitude scale linearly with the mass of the virtual tool. However, while the torque is independent of the orientation of the tool, the force direction rotates with the tool. Using the WristBOT, we could independently control the torques and forces that subjects experienced as well as the visual orientation of the tool.

There are several differences between manipulating a real hammer and the tool simulated here. First, as the simulated tool was constrained to the horizontal plane, there were no effects of gravity. Second, the simulated tool was grasped by a handle which protruded vertically from the plane of rotation, whereas a real hammer is grasped by a handle which is aligned with the long-axis of the tool. These features were important in allowing us to limit sensory feedback about the orientation of the tool to vision and active dynamic interaction.

Experiment 1: Visual Cueing of Dynamics

The first experiment was designed to examine whether vision of a hammer-like tool alone would activate a representation of the appropriate dynamics. Subjects (n=8) grasped the handle of the WristBOT and were presented with the tool at different visual orientations (inset of Figure 1B in the main text). The visual orientation of the tool changed every 4 trials chosen from [0, -45, -90, -135, -180°]. Within a block of 20 trials each of these orientations was experienced once in a pseudo-random order, and 2 blocks were performed giving a total of 40 trials. The WristBOT generated the torques associated with rotating a tool with mass of 400 and 800 g, randomized over the two blocks. Although the visual orientation changed, the WristBOT did not simulate the translational forces associated with the dynamics. Instead, the handle was clamped by a stiff spring (1000 N/m) centred on the handle position at the start of each trial. Therefore, any forces produced by the subject as they rotated the tool were recorded as equal but opposite forces generated by the spring. Such “error clamp” trials minimise kinematic errors and thus minimise any learning (or unlearning) [35], allowing us to assess the anticipatory forces produced by subjects based on their prior knowledge of the expected dynamics. We examined the magnitude (repeated measures ANOVAs) and direction (circular-circular association) of the peak force generated by the subjects as a function of visual orientation of the tool.

Experiment 2: Single-Orientation Exposure and Generalization

The second experiment was designed to examine the effects of exposure to the tool dynamics at a single orientation and the transfer of any associated learning to a novel probe orientation. Subjects (n=9) experienced 7 different tools with different inertial properties. For each tool the forces and torques could be independently varied to be appropriate for a mass that was a factor of 0.7, 1.0 or 1.3 of the base tool mass (1% of the subject’s body mass). The forces and torques could either scale congruently as for real-world objects (3 tools: force:torque = 0.7:0.7, 1.0:1.0, 1.3:1.3) , or incongruently (4 tools: force:torque = 0.7:1.0, 1.3:1.0, 1.0:0.7, 1.0:1.3). Each tool was presented in a separate blocks of 90 trials (pseudo-randomized order) yielding a total of 630 trials. Each block began with an exposure phase of 60 trials during which subjects experienced the tool at an orientation of 0°. In the final 30 trials one error clamp trial was inserted randomly every 5 trials. On these error clamp trials, the torques were generated as usual but the forces associated with the dynamics of the tool were turned off and the handle was held in place by a stiff spring as described above. For these error clamp trials the visual orientation of the tool either remained at 0° or was displayed at -90°. By examining the translational forces generated by the subjects on these error clamp trials we could assess both

learning at the training orientation and transfer to the novel probe orientation. Only data for congruently scaled tools are analysed here.

Experiment 3: Generalization to Multiple Orientations

The third experiment was designed to examine the pattern of transfer to multiple generalization orientations after exposure at a single orientation. Subjects (n=8) were exposed to the tool at 0° for 64 trials. They were then presented with short blocks of eight trials at various probe orientations (0, -22.5, -45, -90 and 180°) followed by a further 18 trials at the training orientation. The first 2 trials immediately following probe trials were error-clamp trials during which the forces produced by subjects were measured. The final 16 trials were under the full dynamics of the tool. Probe orientations were presented in pseudo-randomized order and each of the 5 probe orientations was presented 3 times for each direction of rotation (a total of 30 blocks). During the probe trials the perturbing forces of the tool were turned off. The peak displacement of the handle during the first 2 trials (CCW / CW rotation pair) was used as a measure of the angle and magnitude of the anticipatory forces produced by subjects at each probe orientation. In addition, the increase in peak displacement at the training orientation following partial de-adaptation induced by probe trials (with the forces turned off), was also measured. Specifically, the increase in peak displacement from the 2 trials immediately before the probe (pre-probe) and immediately after the probe (post-probe) was measured. Rest trials were given every 3-5 minutes as in Experiment 2.

Experiment 4: Dissociation of Visual Orientation and Dynamics

The fourth experiment was designed to examine the interaction between the visual and dynamic properties of the tool. To do this we dissociated the visual orientation from the orientation of the tool dynamics. The visual orientation could be either congruent with the dynamics (vision:dynamics at 0°:0° or 180°:180°) or incongruent (vision:dynamics at 0°:180° or 180°:0°). Subjects (n=28) were divided into four groups (of 7 subjects). Two groups experienced a congruent tool, either at 0 or 180° and two groups experienced an incongruent tool, with vision at 0° and dynamics at 180° or vice versa.

Subjects always experienced the torque associated with rotating the tool and experienced the forces on exposure trials. The experiment was performed in 2 parts. In the first part, subjects experienced the tool at the exposure orientation (either 0 or 180°) and completed a total of 320 trials. A block of 48 familiarization trials were given first in which the forces associated with the dynamics were turned off. A block of 224 exposure trials followed immediately in which subjects experienced the full dynamics of the tool. In the final 128 trials of the exposure period one error-clamp trial was inserted randomly every 8 trials for a total of 16 error-clamp trials (8 for each direction, CW and CCW at the exposure orientation). Finally, the subjects performed 48 post-exposure trials during which the forces where again turned off. The second part of the experiment was similar to the first except that error-clamp trials were given at both the exposure orientation (0 or 180°) as well as a probe orientation (-90°). These error-clamp trials were included to probe generalization of the effects of exposure to congruent versus incongruent tools to a novel orientation where the dynamics had not been directly experienced. In this case subjects completed 256 exposure trials during which one error-clamp trial was inserted randomly every 8 trials. A total of 32 error-clamp trials were present (8 for each direction and orientation). Rest trials were given every 3-5 minutes as in experiment 2.

Data Collection and Statistical Analysis

Data was collected from the manipulandum at 1000 Hz and logged to disk for offline analysis using Matlab. Trial-by-trial performance was defined as the maximum displacement (independent of direction) of the handle during the movement, relative to its position at movement onset. On error-clamp trials, the magnitude and direction the peak force produced by subjects was analysed. All angular data were analysed using circular statistical methods [2]. All other statistical tests were performed using Matlab or SPSS (V17.0, SPSS Inc., Chicago, IL, USA). All t-tests were two-tailed.

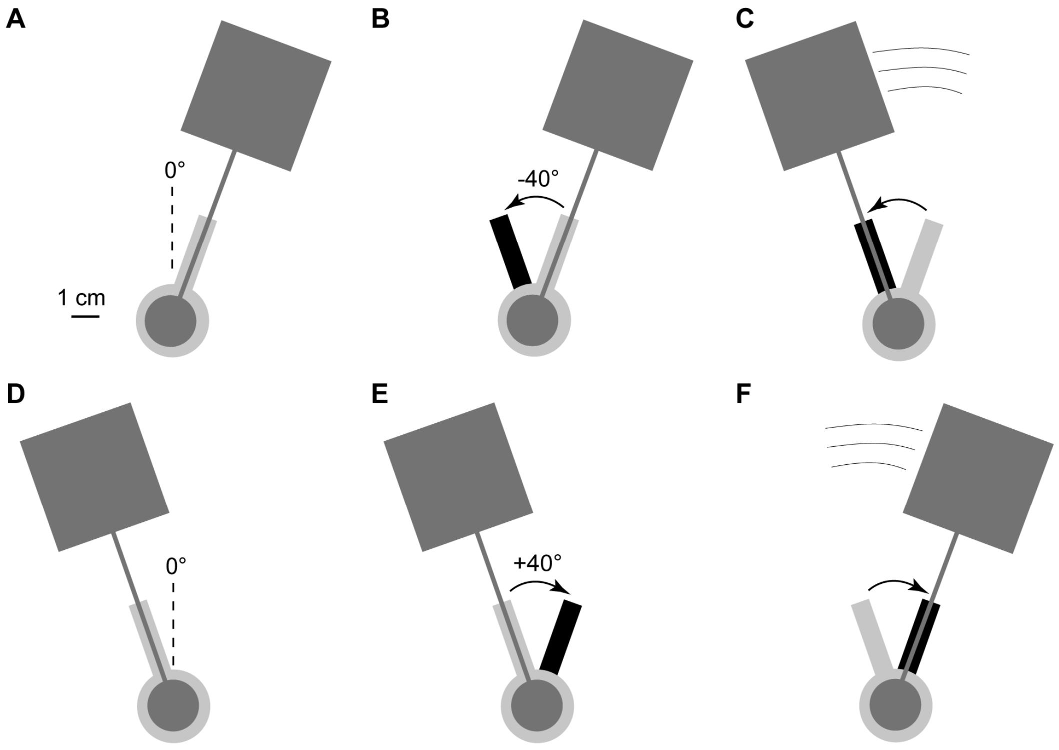

Figure S1. Visual Feedback of the Tool and the Task
Subjects made alternating 40° CCW (A-C) and 40° CW (D-F) rotations of the tool.
(A) A CCW rotation trial began with the tool (dark grey) positioned in the home position (light grey disc) and aligned with the +20° target (light grey bar).
(B) The -20° target for the CCW rotation was visually displayed (black bar) and a tone cued movement onset.
(C) The subject rotated the tool CCW to reach the -20° target within 400 ms.
(D) A CW rotation trial began with the tool (dark grey) positioned in the home position (light grey disc) and aligned with the -20° target (light grey bar).
(E) The +20° target for the CW rotation was visually displayed (black bar) and a tone cued movement onset.
(F) The subject rotated the tool CW to reach the +20° target within 400 ms.
